# Efficacy and safety of pan retinal photocoagulation combined with intravitreal anti-VEGF agents for high-risk proliferative diabetic retinopathy: A systematic review and meta-analysis

**DOI:** 10.1097/MD.0000000000034856

**Published:** 2023-09-29

**Authors:** Peng Fu, Yanling Huang, Xiaobo Wan, Huiyi Zuo, Yong Yang, Renshen Shi, Minli Huang

**Affiliations:** a Department of Ophthalmology, the First Affiliated Hospital of Guangxi Medical University, Nanning, Guangxi, China; b Department of Ophthalmology, Liuzhou People’s Hospital affiliated to Guangxi Medical University, Liuzhou, Guangxi, China.

**Keywords:** anti-vascular endothelial growth factor, efficacy and safety, high-risk proliferative diabetic retinopathy, meta analysis, pan retinal photocoagulation

## Abstract

**Background:**

High-risk proliferative diabetic retinopathy (HR-PDR) is the advanced stage of diabetic retinopathy progression with poor prior treatment efficacy and high rates of blindness. This meta-analysis aims to compare the efficacy and safety of pan retinal photocoagulation (PRP) combined with intravitreal anti-vascular endothelial growth factor (aVEGF) (PRP + aVEGF) versus PRP monotherapy in HR-PDR patients.

**Methods:**

A thorough search was performed through PubMed, Web of Science, EMBASE, and the Cochran Library from inception to December 18, 2022. Outcome measures included change in central macular thickness, best-corrected visual acuity, fluorescein angiography, incidence of undergoing vitrectomy, and adverse events during the follow-up period.

**Results:**

Eight studies (6 randomized controlled trials and 2 retrospective studies) with 375 eyes were included in this meta-analysis. There were no obvious differences in the changes of best-corrected visual acuity and fluorescein angiography between the PRP + aVEGF and PRP monotherapy groups. However, PRP + aVEGF group had a significant reduction in the change of central macula thickness (standard mean deviations = −1.44, 95%CI = −2.55 to −0.32, *P* = .01) and the rate of undergoing vitrectomy (odds ratio = 0.20, 95%CI = 0.05–0.83, *P* = .01). Additionally, the risks of vitreous hemorrhage and other complications were not significantly different between the 2 groups.

**Conclusion subsections:**

Our meta-analysis indicated that PRP + aVEGF might have potential benefits in the treatment of HR-PDR patients. However, given several limitations of this study, more research is needed to confirm our findings.

## 1. Introduction

Diabetic retinopathy (DR) is a severe ocular microvascular complication of diabetes mellitus, which is the leading cause of visual impairment in the working-age population.^[1–3]^ DR is significantly associated with future risk of congestive heart failure, myocardial infarction and cerebrovascular accident.^[4]^ The global prevalence of DR was as high as 27% between 2015 and 2018.^[5]^ Around 10% of the entire diabetic population, and one-third of DR patients, are likely to progress to the stage of diabetic macular edema (DME) or proliferative diabetic retinopathy (PDR).^[6]^ DR is considered the leading cause of vision loss, mainly due to pathological neovascularization and DME.^[7,8]^

High-risk proliferative diabetic retinopathy (HR-PDR) was defined in accordance with the early treatment diabetic retinopathy study research group guidelines^[9]^ as follows: Neovascularization of the disc more than 1/4 disc area of the optic disc; preretinal or vitreous hemorrhage due to Neovascularization of the disc; Neovascularization elsewhere more than a half disc area, along with preretinal or vitreous hemorrhage. HR-PDR is the advanced stage of DR progression with poor prior treatment efficacy and high rates of blindness. In the landmark DR study, 44.0% of patients with HR-PDR experienced a significant loss of visual function after 4 years.^[10]^ Thus, the increased severity of HR-PDR can aggravate PDR progression and lead to vision loss.

Vascular endothelial factor (VEGF) has been implicated in the neovascularization of the human eye and is an important factor for the progression of DR.^[11]^ Currently, the standard treatment options for DR include intravitreal injection of anti-vascular endothelial growth factor (aVEGF) agents, panretinal photocoagulation (PRP), and vitrectomy.^[12]^ aVEGF agents show promise in treating patients with PDR or DME.^[13]^ PRP is effective in reducing oxygen demand in the retina and preventing the development of neovascularization.^[14]^ It has been used for the treatment of DR for over 4 decades,^[15]^ and has been shown to decrease the likelihood of experiencing significant visual loss by approximately 50%.^[16]^ However, it is important to note that PRP treatment is associated with several side effects due to its destructive nature. These side effects include reduced peripheral visual field, color vision disturbances, night vision problems, lower contrast sensitivity, macular edema, pain during the procedure, phlebotomy syndrome, etc.^[17]^ Additionally, in some cases (approximately 4.5%), sever vitreous hemorrhage may occur, necessitating pars plana vitrectomy for resolution.^[18]^ aVEGF treatment often requires repeated administration as the duration of its effects is short. Despite intervention, the damage to the retina caused by fibrosis and shrinkage in PDR may be irreversible, which in turn leads to long-term consequences, including severe visual impairment. Therefore, preventing the progression of DME and PDR is a key objective for public health initiatives.

Vision loss may occur as a consequence of microvascular ischemia related to DR. A previous study reported that retinal ischemia occurred in patients with HR-PDR,^[19]^ proactive treatment of HR-PDR could delay the progression of vision-threatening complications; and less extensive PRP was required to manage HR-PDR when the combination of PRP and aVEGF was applied. However, the effects of PRP combined with aVEGF in treating HR-PDR are still controversial.

During the last decade, numerous randomized controlled trials (RCTs) and retrospective studies have analyzed and compared the efficacy of PRP combined with intravitreal aVEGF (PRP + aVEGF) and PRP monotherapy in the management of HR-PDR. However, there are still conflicting opinions on the efficacy and safety of these 2 therapies. This meta-analysis aimed to verify the efficacy and safety of PRP combined with intravitreal aVEGF therapy versus PRP monotherapy in HR-PDR patients.

## 2. Methods

### 2.1. Database and search strategy

A thorough search was conducted in 4 databases (Cochrane Library, EMBASE, Web of Science and PubMed) from inception to December 18, 2022. The search terms included (“diabetic retinopathy” OR “diabetic retinopathies” OR “proliferative diabetic retinopathy” OR PDR) AND (“retinal laser photocoagulation” OR photocoagulation OR “laser photocoagulation”) AND (“vascular endothelial growth factor” OR VEGF OR anti-VEGF OR ranibizumab OR lucentis OR bevacizumab OR avastin OR aflibercept OR eylea). Only the articles published in English and in peer-reviewed journals were retrieved, with no restrictions on publication status or date. The references of previous reviews and included studies of this meta-analysis underwent a thorough manual screening process to ensure that no eligible studies were overlooked, which were not previously identified.

### 2.2. Inclusion and exclusion criteria

The article was screened according to the following criteria: The patients should be clinically diagnosed HR-PDR; The final articles should be cohort studies or RCTs, comparing the safety and efficacy of PRP + aVEGF with PRP monotherapy in HR-PDR patients; The extracted data must contain central macular thickness (CMT) or best-corrected visual acuity (BCVA); and The follow-up period should be at least 3 months. The exclusion criteria were as follows: studies with less than 5 patients in each group; studies that were post hoc analyses of RCTs; studies without complete data of the main outcomes, as well as review articles, case reports or case series, were excluded.

### 2.3. Literature screening, data extraction and study quality assessment

Two investigators independently reviewed titles and abstracts together according to the inclusion and exclusion criteria, and retrieved suitable articles for full-text evaluation. Disagreement was resolved by discussion with a third reviewer to reach consensus. Data were obtained in a standardized format from each of the included studies. The recorded data variables were the authors, publication year, country of origin, number of patients, age, clinical outcomes and follow-up period. The efficacy outcomes included the change in BCVA, CMT, fluorescein angiography (FLA) and incidence of vitrectomy. The primary efficacy outcomes included BCVA and CMT, while FLA and the incidence of vitrectomy were considered as the secondary efficacy outcomes. The safety outcomes included the vitreous hemorrhage, intraocular pressure elevation, adverse events (AEs) and so on. The risk of bias for each included RCT was evaluated using the Cochrane Collaboration tool.^[20]^ For non-randomized studies, the risk of bias was assessed using the Risk of Bias in Non-randomized Studies of Interventions tool.^[21]^ Risk of bias was examined by 2 independent authors (Peng Fu and Yanlin Huang). Conflicts were resolved in consultation with a third independent author (Minli Huang).

### 2.4. Statistical methods

Statistical tests were conducted with RevMan 5.4 software. Dichotomous data were recorded using the odds ratios (ORs) and its 95% confidence intervals (CIs), while continuous data were recorded using the standard mean deviations (SMDs) and its 95% CIs for effect size statistics. When studies reported only early treatment diabetic retinopathy study research group letters, the values were converted to LogMAR-BCVA data for analysis using the methods suggested by Khoshnood and coworkers.^[22]^ To reveal the characteristics of each group, the effect estimates were pooled using a random-effect model. *P* < .05 was deemed statistically significant. The statistical heterogeneity among trials was measured by Q statistics and the *I*^2^ test, where *I*^2^ > 50% or *P* < .1 indicated heterogeneity of results.^[23]^ When > 10 studies were identified, the risk of publication bias was assessed using a funnel plot of the obtained effect estimates.

## 3. Results

### 3.1. Literature screening

**Figure 1.**
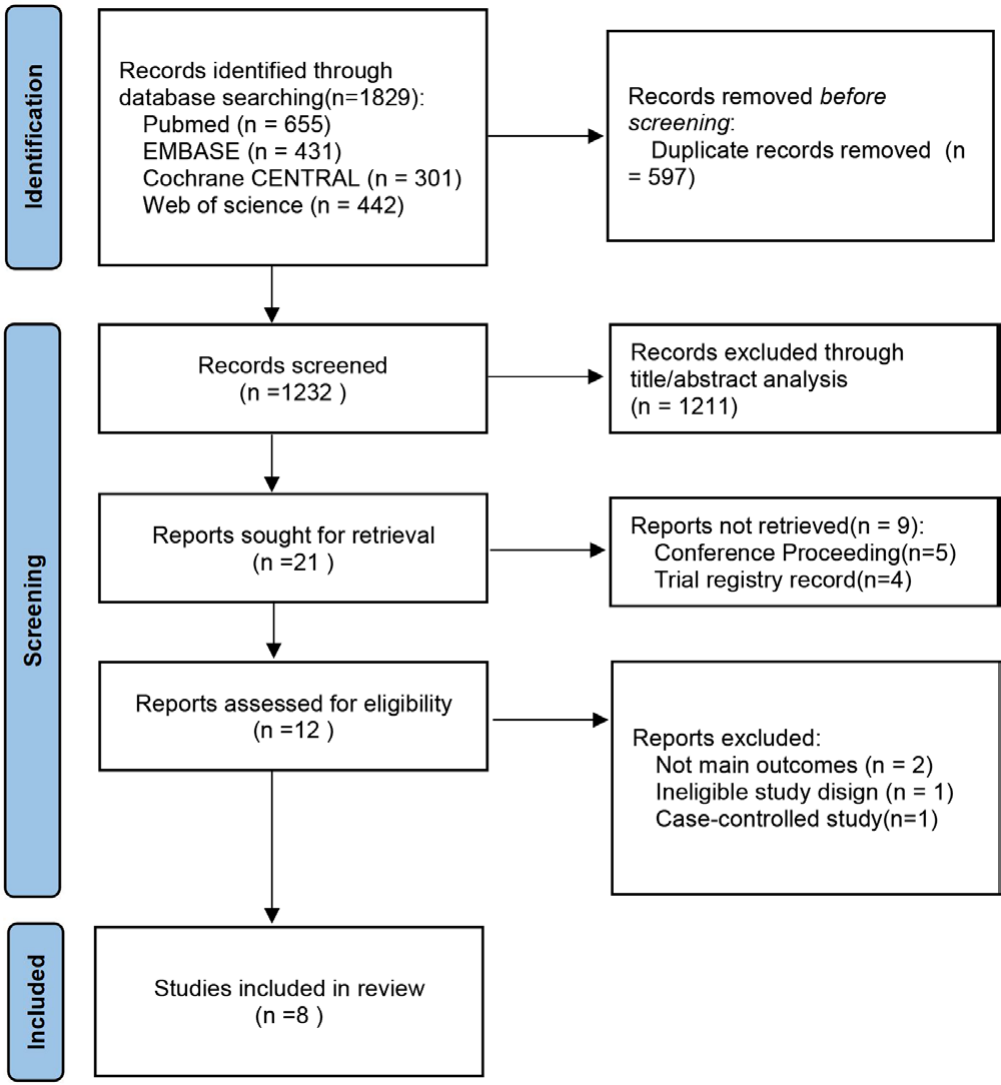
PRISMA flowchart of the included studies.

Figure 1 is a flow diagram presenting an overview of the literature selection process. In total, 1829 studies were identified, after retrieving 21 articles for full-text review, 13 articles were excluded because of conference proceeding (n = 5), trial registry record (n = 4), not main outcomes (n = 2), ineligible study design (n = 1), and case-controlled study (n = 1). Eight studies (published from January 2008 to March 2021), with 375 eyes, were included in this quantitative meta-analysis.

**Figure 2.**
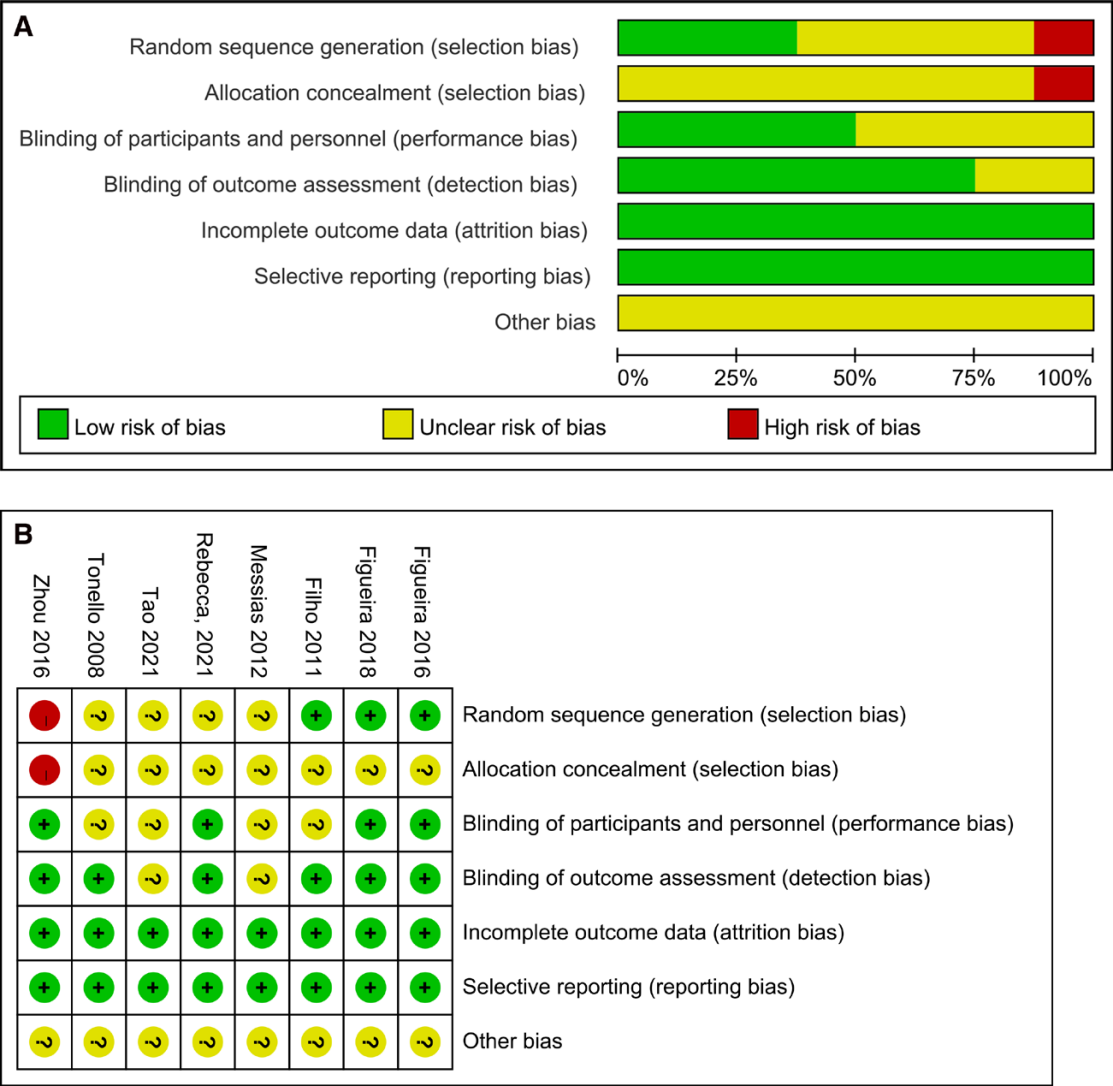
Risk of bias.

Overall, the 8 studies corresponded to 6 RCTs^[24–29]^ and 2 retrospective studies.^[30,31]^ There were 3 studies compared PRP plus intravitreal bevacizumab with PRP monotherapy, 4 studies compared PRP plus intravitreal ranibizumab with PRP monotherapy, and 1 study compared PRP plus intravitreal aflibercept with PRP monotherapy. The mean age of patients was 56.36 ± 9.51 years, and 40.52% were female. The range of follow-up duration was 3 to 12 months. Further details of trial designs are included in Table 1. The risk of bias was evaluated for the 6 RCTs (Fig. 2A and B). The majority of RCTs were assessed as having a moderate risk of bias; and 1 RCT exhibited a high risk of bias. Regarding the retrospective studies, one of them was deemed to have a moderate risk of bias, while the other retrospective study was determined to have a high risk of bias (Supplemental Table 1, http://links.lww.com/MD/J620).

**Table 1:** Characteristics of included studies.

Included studies	Location	Study Design	Interventions	Sample size (eyes)	Average age (SD)	Famle (%)	Outcomes	Follow-up
Figueira et al (2016)	America	RCT	PRP combined Ranibizumab	12	57.0 (8.9)	2 (16.7)	BCVA,CMT,AEs	12 mo
			PRP	13	54.0 (11.1)	3 (23.1)		
Figueira et al (2018)	America	RCT	PRP combined Ranibizumab	41	58.8 (13.3)	13 (31.7)	BCVA,CMT,AEs	12 mo
			PRP	46	52.0 (11.9)	19 (41.3)		
Filho et al (2011)	America	RCT	PRP combined Ranibizumab	15	50.5 (11.6)	6 (40.0)	FLA,BCVA,CSMT, AEs	12 mo
			PRP	14	63.3 (9.4)	5 (35.7)		
Messias et al (2012)	Brazil	RCT	PRP combined Ranibizumab	11	59.0 (12.0)	5 (45.5)	BCVA,FLA	12 mo
			PRP	9	64.0 (8.0)	4 (44.4)		
Rebecca et al (2021)	Pakistan	RCT	PRP combined Bevacizumab	38	51.1 (5.9)	NR(37.0)	BCVA,CMT	12 mo
			PRP	38	50.7 (6.9)	NR(41.8)		
tao et al (2021)	China	Retrospective	PRP combined Aflibercept	40	62.7 (6.8)	20 (55.6)	BCVA, CFT,AEs	6 mo
			PRP	32	61.4 (7.1)	9 (56.3)		
Tonello et al (2008)	America	RCT	PRP combined Bevacizumab	15	54.1 (11.7)	4 (26.7)	BCVA,AEs	4 mo
			PRP	15	51.4 (10.7)	7 (46.7)		
Zhou et al (2016)	China	Retrospective	PRP combined Bevacizumab	18	53.9 (8.1)	9 (50.0)	BCVA,CSMT,FLA,AEs	12 mo
			PRP	18	57.9 (8.7)	7 (38.9)		

AEs = adverse event, BCVA = best-corrected visual acuity, CFT = central foveal thickness, CMT = central macula thickness, CSMT = central subfield macular thickness, FLA = fluorescein angiography, NR = not reported, PRP = panretinal photocoagulation, RCT = randomized controlled trials.

### 3.2. The efficacy outcomes

#### 3.2.1. The primary efficacy outcomes.

##### 3.2.1.1. Changes in BCVA.

**Figure 3.**
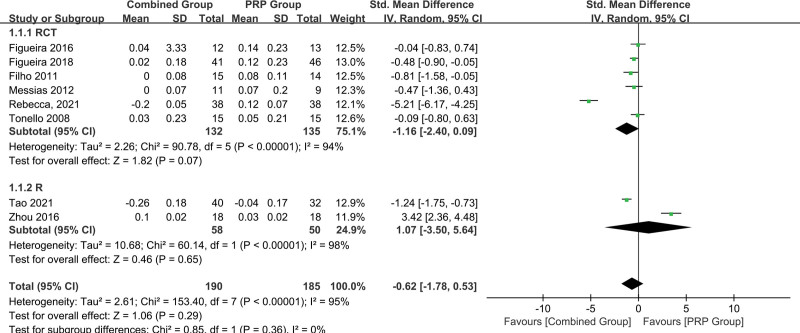
Subgroup analysis of randomized controlled trials (RCTs) and retrospective studies on the BCVA (logMAR) changes between the PRP + aVEGF and PRP monotherapy groups. aVEGF = anti-vascular endothelial growth factor, BCVA = best-corrected visual acuity.

Six RCTs^[24–29]^ and 2 retrospective studies^[30,31]^ reported the outcome of BCVA changes, of which 6 studies reported BCVA changes in logMAR and 2 studies in letters. All visual acuity data were transformed into logMAR prior to statistical analysis. The pooled results (Fig. 3) demonstrated that BCVA gain was not significantly different between the PRP + aVEGF and PRP monotherapy groups in 6 RCT studies (SMD = −1.16, 95%CI = −2.40–0.09, *P* = .07) and 2 retrospective studies (SMD = 1.07, 95%CI = −3.50–5.64, *P* = .65).

##### 3.2.1.2. Changes in macular thickness.

**Figure 4.**
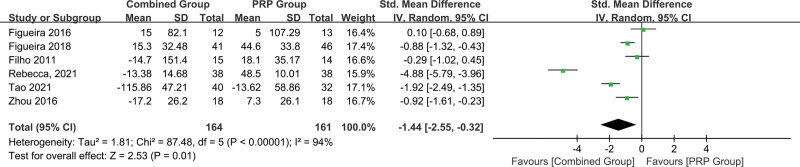
Forest plot summarizing anatomic outcomes. The changes of macular thickness between the 2 groups.

Six studies^[24–26,28,30,31]^ reported the changes of macular thickness from baseline as anatomic outcomes with 3 studies^[24,25,28]^ reported CMT, 2 studies^[26,31]^ reported as central subfield macular thickness and 1 study^[30]^ reported as central foveal thickness. This meta-analysis (Fig. 4) demonstrated a significant reduction in macular retinal thickness changes between the PRP + aVEGF and PRP monotherapy groups (SMD = −1.44,95%CI = −2.55 to −0.32, *P* = .01).

#### 3.2.2. The secondary efficacy outcomes.

##### 3.2.2.1. Changes in FLA.

**Figure 5.**
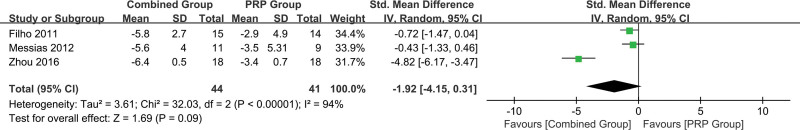
Forest plot demonstrating the changes of fluorescein angiography (FLA) between the 2 groups.

The FLA was reported in 3 studies^[26,27,31]^ (Fig. 5), and no obvious difference in FLA was found between the PRP + aVEGF and PRP monotherapy groups (SMD = −1.92,95%CI = −4.15–0.31, *P* = .09).

##### 3.2.2.2. Incidence of vitrectomy.

**Figure 6.**
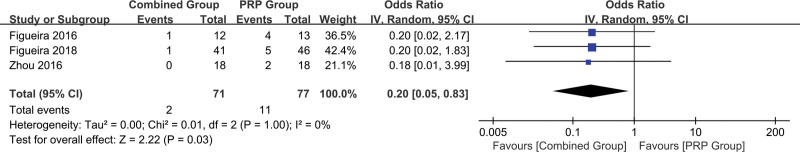
Forest plot demonstrating the incidence of vitrectomy between the 2 groups.

Three studies^[24,25,31]^ reported that a proportion of patients underwent vitrectomy due to vitreous hemorrhage, tractional retinal detachment or other DR complications during follow-up time. As shown in Figure 6, there was statistically significant difference between PRP + aVEGF and PRP monotherapy group (OR = 0.20, 95%CI = 0.05–0.83, *P* = .01).

### 3.3. The safety outcomes

**Figure 7.**
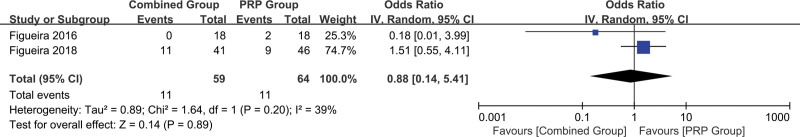
Forest plot demonstrating the incidence of AEs (vitreous hemorrhage) between the 2 groups. AEs = adverse events.

Two studies^[24,31]^ reported Vitreous hemorrhage, 2 studies^[24,31]^ reported intraocular pressure elevation, cataract, subconjunctival hemorrhage and anterior uveitis, 2 studies^[25,29]^ reported no AEs, and 2 studies^[27,28]^ without reported AEs. The results showed that no obvious difference was found in the risk of vitreous hemorrhage between the PRP + aVEGF and PRP monotherapy groups (OR = 0.88, 95%CI = 0.14–5.41, *P* = .89) (Fig. 7).

### 3.4. Analysis of publication bias

The funnel plot was not reported, as only 8 studies were included in this meta-analysis.

## 4. Discussion

Vision loss associated with DR is most commonly attributed to DME, and VEGF has a central implication in the pathogenesis of DME.^[32]^ At present, the effects of aVEGF in HR-PDR have not been extensively investigated.^[31]^ There are still conflicting opinions about PRP combined with aVEGF for the management of HR-PDR patients, especially in those without clinically significant macular edema (CSME). Once PDR or complicated DME has developed, the extent of the damage to the retina may be irreversible. Thus, it is important to explore earlier and more effective therapies for the treatment of HR-PDR. The combination of PRP and aVEGF at the earlier stage may lead to unexpected therapeutic outcomes. Yang et al^[33]^ found that the combination of intravitreal bevacizumab and PRP demonstrated favorable short-term anatomical and functional outcomes in treating HR-PDR-associated complications. Combination therapy with PRP and ranibizumab showed a potential benefit of preserving contrast sensitivity compared to PRP monotherapy in patients without HR-PDR.^[34]^ The present study demonstrated that PRP + aVEGF was superior to PRP monotherapy in treating HR-PDR patients. Although there were no significant differences in the change of BCVA and FLA as well as the risks of vitreous hemorrhage and other complications between the 2 groups, the change of CMT and rate of undergoing vitrectomy were markedly decreased in PRP + aVEGF group.

In this meta-analysis, no significant changes in BCVA were detected between the PRP + aVEGF and PRP monotherapy groups after treatment of patients with HR-PDR. This result is inconsistent with a previous meta-analysis performed by Zhang et al^[35]^ In their meta-analysis involving 11 studies, they found that PRP combined with aVEGF could improve BCVA and achieved the desired efficacy in the treatment of Dr However, they included patient with all stage of DR, such as PDR, DME, and HR-PDR. Considering that HR-PDR is the advanced stage of DR progression with poor prior treatment efficacy and high rates of blindness, our meta-analysis was limited to patients with HR-PDR. Hence, our meta-analysis provides more pertinent and accurate evidence concerning the effect of PRP + aVEGF on BCVA in HR-PDR patients. It is possible that HR-PDR patients without CSME have a relatively stable vision throughout the course of the disease. Intravitreal aVEGF may serve as a promising adjunctive therapy to PRP in treating HR-PDR patients, especially those without CSME.^[36]^

However, no obvious difference in FLA was found between the PRP + aVEGF and PRP monotherapy groups in this meta-analysis. Longer follow-up and larger sample studies are needed to confirm the efficacy of combined treatment for the attenuation of FLA in HR-PDR patients.

DME is known to cause permanent visual impairment in DR patients, but the use of focal or grid laser photocoagulation to the macula can significantly reduce the risk of DME by 50%.^[37]^ In recent years, intravitreal antiangiogenic drug injections have been proposed in an attempt to improve visual acuity in patients with DR-associated macular edema.^[38]^ According to the 2017 Global Trends in Retina survey, approximately 28.4% of retina specialists incorporate a combination of PRP and aVEGF for the management of HR-PDR patients without macular edema.^[39]^ Our analysis showed that PRP combined with aVEGF was superior to PRP monotherapy in reducing the thickness of CMT among HR-PDR patients. This result is consistent with previous research showing that CMT is significantly decreased in PRP and aVEGF group.^[36]^

Approximately 4.5% patients treated with PRP progress to require vitrectomy surgery.^[40]^ aVEGF remarkably improves the clearance of vitreous hemorrhage, thus avoiding vitrectomy surgery in HR-PDR patients.^[33]^ Researchers observed that aVEGF injection, followed by standard PRP, could achieve a longer-lasting impact on preventing neovascular recurrence and reduce the AEs of repeated intravitreous injections. Hence, the combination of PRP and aVEGF holds promise in the management of HR-PDR without CSME.^[39]^ PRP combined with aVEGF enables the application of less extensive PRP to manage HR-PDR, thereby minimizing the associated functional loss of the retina.^[27,41]^ Our study reveals that the combined therapy of PRP and aVEGF can be more effective to delay the progression of DR and decrease the risk of vitrectomy. Hence, our findings indicated PRP + aVEGF could be as a promising therapy for HR-PDR patients.

## 5. Limitation

The main limitation of this meta-analysis are the small sample size and short follow-up duration. Only the studies published in English language were included, and some of the studies were retrospective in nature. Hence, the results of this analysis might have a very low certainty of evidence. Further validation with larger sample-sized, multicenter, and low-bias RCTs is needed in the near future. In addition, we were unable to conduct a subgroup analysis of OCT devices due to the inclusion of studies that utilized a variety of OCT devices with differing specifications. It is also worth mentioning that our meta-analysis included only 8 studies. As a result, we were unable to assess the risk of publication bias using a funnel plot, which is a commonly used method to detect potential bias in the reporting of study results. This limitation might have an impact on the reliability and generalizability of our findings.

## 6. Conclusion

Our study indicated that in comparison to PRP monotherapy, PRP + aVEGF could attenuate the central macular thickness, decrease the risk of vitrectomy and prevent further progression of HR-PDR to severe PDR. However, the overall changes in BCVA exhibited no significant difference between 2 groups. In terms of safety outcomes, PRP + aVEGF treatment did not increase the risks of vitreous hemorrhage and other complications. Thus, PRP + aVEGF might have potential benefits in the treatment of HR-PDR patients. Nevertheless, given several limitations of this study, more studies are warranted to verify our findings.

## Acknowledgments

The authors thank all individuals for their participation in this study. The authors would like to express their gratitude to EditSprings (https://www.editsprings.cn) for the expert linguistic services provided.

## Author contributions

**Conceptualization:** Peng Fu, Minli Huang.

**Data curation:** Peng Fu, Yanling Huang.

**Funding acquisition:** Xiaobo Wan.

**Formal analysis:** Peng Fu, Huiyi Zuo.

**Methodology:** Peng Fu, Yong Yang.

**Project administration:** Peng Fu.

**Resources:** Peng Fu.

**Software:** Peng Fu, Renshen Shi.

**Supervision:** Xiaobo Wan, Minli Huang.

**Validation:** Minli Huang.

**Visualization:** Minli Huang.

## Supplementary Material


