# Supplementary material for: Efficacy and safety of pan retinal photocoagulation combined with intravitreal anti-VEGF agents for high-risk proliferative diabetic retinopathy: A systematic review and meta-analysis
Source: Medicine (Baltimore). 2023 Sep 29;102(39):e34856. doi: 10.1097/MD.0000000000034856 (PMC10545261; doi:10.1097/MD.0000000000034856)
Supplement: Supplementary file 1 [file medi-102-e34856-s001.docx]

Supplemental Table 1 Risk of bias assessment in the included studies using Risk of Bias in Non-randomized Studies of Interventions (ROBINS-I) tool

| Study/Bias domain | Tao 2021 | Zhou 2016 |
| --- | --- | --- |
| Bias due to confounding | Moderate risk of bias | Serious risk of bias  Not adjusted for age, sex, etc. |
| Bias in selection of participants into the study | Low risk of bias | Low risk of bias |
| Bias in classification of interventions | Low risk of bias | Low risk of bias |
| Bias due to deviations from intended interventions | Low risk of bias | Low risk of bias |
| Bias due to missing data | No Information | No Information |
| Bias in measurement of outcomes | Low risk of bias | Low risk of bias |
| Bias in selection of the reported result | Low risk of bias | Low risk of bias |
| Overall | Moderate risk | Serious risk of bias |
